# Role of tomography in the screening at the emergency room during pandemics

**DOI:** 10.1016/j.clinsp.2024.100559

**Published:** 2024-12-12

**Authors:** Marcel Lima Albuquerque

**Affiliations:** Hospital Cárdio Pulmonar, Escola Bahiana de Medicina e Saúde Pública, Salvador, BA, Brazil

**Keywords:** Tomography, COVID-19, Pandemics

Acute respiratory pandemics are cyclical in humanity's history, and tools that confirm etiological diagnosis take time to emerge or do not provide immediate results.^1^ In COVID-19, the gold standard test for diagnosis is the detection of viral genetic material through the Polymerase Chain Reaction (RT-PCR).^2^ Other more available methods can be used to assess the likelihood of the diagnosis more promptly. Therefore, the use of chest Computed Tomography (CT), a more available exam with immediate results, can assess the likelihood of COVID-19 diagnosis and other respiratory infections.

The authors conducted a retrospective cohort study involving patients over 18 years old who attended for suspected COVID-19 in the Emergency Room (ER) in a private hospital which integrated the registry for the management of suspected or confirmed patients by COVID-19 (RECOVID-BA), during the COVID-19 pandemic period from April to August 2020. Patients ≥ 18 years old who searched for medical care in the ER mainly due to acute respiratory symptoms and performed both CT and RT-PCR for SARS-CoV2 were included in the study.

Acute COVID-19 infection was assumed if an acute onset of respiratory symptoms and a positive RT-PCR for SARS-CoV2 were present in the subjects studied. Patients were then divided into two groups: those with a positive RT-PCR or a negative RT-PCR. Chest CT was performed in all subjects with clinical suspicion of acute COVID, but CT reports from radiologists were produced in a blind environment analysis according to SARS-CoV2 RT-PCR status.

A suggestive CT pattern of acute viral pneumonia according to the radiologist's opinion was defined as the presence of peripheral, focal, or multifocal ground-glass opacities, bilateral involvement and mosaic attenuation pattern.

The description of the radiological pattern was recorded according to both American and Brazilian Radiology College guidelines, including the laterality and percentage of lung involvement.^3^^,^^4^

The frequency of positive RT-PCR tests, the sensitivity, specificity, Positive and Negative Predictive Values (PPV and NPV), and also accuracy were calculated using the result (positive or negative RT-PCR status) as a reference. A convenience sample was used. This study was approved by the local ethical committee (CAAE n° 30,564,420.1.1001.0008).

The majority (66.1 % or 497 subjects) of the 751 patients included in the study had a positive RT-PCR, assumed as having an acute respiratory SARS-CoV2 infection. Gender and age distribution, smoking status, other comorbidities frequency, and the chest CT findings are presented in Table 1.

**Table 1:** Clinical and demographic characteristics of the total population and characteristics of chest tomography patterns.

Variables	Total (*n* = 751)	COVID-19 NO (*n* = 254)	COVID-19 YES (*n* = 497)	p
Age (IQ)	60 years old (31)	61.00 (38)	59.00 (31)	0.899
Male, n (%)	432 (57.5 %)	131 (51.6 %)	301 (60.6 %)	0.027
Hypertension, n (%)	343 (45.7 %)	111 (43.7 %)	232 (46.7 %)	0.438
Diabetes n (%)	153 (20.8 %)	43 (16.9 %)	113 (22.7 %)	0.063
COPD, n (%)	30 (4.0 %)	12 (5.1 %)	18 (6.6 %)	0.465
Kidney failure, n (%)	46 (6.1 %)	13 (5.1 %)	33 (6.6 %)	0.411
Asthma, n (%)	34 (4.5 %)	18 (7.1 %)	16 (3.2 %)	0.016
Smoking, n (%)	16 (2.1 %)	9 (3.5 %)	7 (1.4 %)	0.055
Obesity, n (%)	76 (10.1 %)	12 (4.7 %)	64 (12.9 %)	0.001
Ground-glass opacity, n (%)	430 (57.3 %)	115 (45.2 %)	315 (63.4 %)	0.001
Bilateral involvement, n (%)	487 (64.8 %)	126 (49.6 %)	361 (72.6 %)	0.001
Suggestive CT pattern, n (%)	461 (61.4 %)	89 (35 %)	372 (74.8)	0.001
Involvement > 50 %	257 (34.2 %)	38 (14.9 %)	219 (44.0 %)	0.001

COPD, Chronic Obstructive Pulmonary Disease.

Using the positive RT-PCR as a reference, the finding of a Suggestive CT pattern of acute viral pneumonia had a 74.8 % sensitivity, a 65 % specificity, and PPV and NPV were, respectively, 80.7 % and 56.9 %, assuming a prevalence of 66.1 % acute respiratory SARS-CoV2 infection found in the present study. A more detailed analysis of the types and laterality of CT patterns found are described in Fig. 1.

**Fig. 1:**
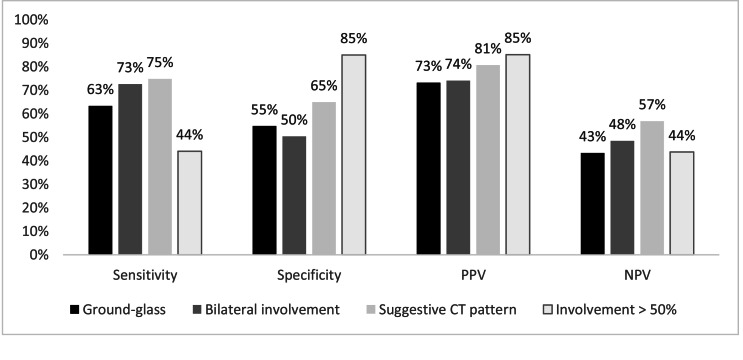
Sensitivity, specificity, positive and negative predictive value of chest tomography patterns. PPV, Positive predictive value; NPV, Negative predictive value.

The majority of subjects (67.8 %, *n* = 509) had to be hospitalized due to the severity of illness, and 36.8 % (*n* = 276) required supplemental oxygen support and/or noninvasive mechanical ventilation (either by low-flow catheters, oxygen masks, or high-flow catheters, and even non-invasive or invasive mechanical ventilation). Of the patients who needed oxygen supplementation, 72 (26 %) ruled out the diagnosis of COVID-19 with a negative RT-PCR test.

Overall mortality was 9.7 % (73 subjects), and mortality in COVID YES and in COVID NO groups were, respectively, 10.9 % and 7.5 %.

Respiratory viral pandemics are cyclical in humanity's history^1^^,^^5^^,^^6^ and this paper adds some relevant data not only to the recent past COVID-19 pandemic, but also for future respiratory viral pandemics that may reach humanity in the future. The accuracy, sensitivity, and specificity, together with PPV and NPV values of chest CT results obtained in the present study (conducted in between a pandemic pattern of a 66.1 % COVID-19 prevalence) reinforces its relevance in supporting clinical decisions in the ER department, which may be useful in the next respiratory pandemic, in the future. The assessment of the radiologist with the conclusion of the examination suggestive of viral infection had adequate accuracy with good specificity and sensitivity, as well as good positive predictive value. The presence of ground-glass opacity together with bilateral involvement was more prevalent in COVID-19 positive patients, revealing its good sensitivity, but at a cost of lower specificity in ruling out COVID-19. The involvement greater than 50 % on chest CT was three times more frequent in COVID-19 patients, a specific finding considered useful in a clinical decision pandemic scenario.

In the practical clinical scenario of the COVID pandemic, sometimes there was some delay in confirming COVID-19 RT-PCR status in the ER, reinforcing the value of chest tomography (which sometimes was faster to obtain than the result of the RT-PCR) results in supporting diagnostic suspect by physicians, as well as helping to better tailoring of the clinical pathways of therapeutic itineraries into the hospital, especially in those with higher severity of the disease.

The pattern of frequent extensive and bilateral pulmonary findings was more frequent in patients with worse outcomes as use the of mechanical ventilation and death. Some scoring systems in the literature have been developed in an attempt to standardize this quantitative analysis; however, there are several limitations, such as the limited number of patients evaluated in previous studies, as well as the lack of correlation with other risk factors (clinical and laboratory) and with histopathological findings. Some models for assessing the extent of pulmonary involvement in diffuse pulmonary diseases are visual/semiquantitative and quantitative. The experience developed in the last decade mainly refers to chronic diseases such as fibrosing pneumopathies and pulmonary emphysema and may be transposable to infectious involvement.^7^^,^^8^

Although chest CT scans involve exposure to ionizing radiation, the statistical risk associated with these procedures remains relatively low, especially when performed at recommended intervals. Advances in CT technology, including low-dose protocols, have further reduced radiation exposure, making it a safer option for diagnostic and monitoring purposes. While cumulative exposure from frequent scans could pose long-term risks, these are minimal for most patients, particularly when balanced against the diagnostic benefits that CT scans provide. For patients requiring regular monitoring, maintaining a reasonable interval between scans and using alternative imaging methods when appropriate can optimize both safety and diagnostic efficacy.^9^

In conclusion, Chest CT scans have emerged as crucial diagnostic tools in the management of COVID-19, offering rapid and effective visualization of lung involvement that correlates with disease severity. This study highlights the potential of integrating radiological assessments with clinical data to enhance the accuracy of COVID-19 diagnoses. Future research should focus on refining the diagnostic criteria based on CT imaging and exploring the role of advanced imaging techniques in the ongoing pandemic management.

## Declaration of competing interest

The authors declare no conflicts of interest.
